# Expression of Concern: miR-26a-5p mediates TLR signaling pathway by targeting CTGF in LPS induced alveolar macrophage

**DOI:** 10.1042/BSR-2019-2598_EOC

**Published:** 2022-11-11

**Keywords:** connective tissue growth factor, miR-26a-5p, severe pneumonia, toll-like receptor signaling pathway

The Editorial Office has been made aware of potential issues surrounding the scientific validity of this paper, hence has issued an expression of concern to notify readers whilst the Editorial Office investigates. It has been noted that the ‘Model’ line in the flow cytometry data from Figure 5B would be impossible to attain, as it should not bend to the left in such a way when the X value is increasing

